# Fruit waste: a current perspective for the sustainable production of pharmacological, nutraceutical, and bioactive resources

**DOI:** 10.3389/fmicb.2023.1260071

**Published:** 2023-10-24

**Authors:** Shankar Prasad Sha, Debabrata Modak, Sourav Sarkar, Sudipta Kumar Roy, Sumit Prasad Sah, Kriti Ghatani, Soumen Bhattacharjee

**Affiliations:** ^1^Food Microbiology Laboratory, Department of Botany, Kurseong College, Kurseong, India; ^2^Cell and Molecular Biology Laboratory, Department of Zoology, University of North Bengal, Raja Rammohunpur, India; ^3^Food Microbiology Laboratory, Department of Food Technology, University of North Bengal, Raja Rammohunpur, India

**Keywords:** fruit wastes, bioactive compounds, nutraceuticals, diseases, secondary metabolites, pharmacological potential

## Abstract

Fruits are crucial components of a balanced diet and a good source of natural antioxidants, that have proven efficacy in various chronic illnesses. Various kinds of waste generated from fruit industries are considered a global concern. By utilizing this fruit waste, the international goal of “zero waste” can be achieved by sustainable utilization of these waste materials as a rich source of secondary metabolites. Moreover, to overcome this waste burden, research have focused on recovering the bioactive compounds from fruit industries and obtaining a new strategy to combat certain chronic diseases. The separation of high-value substances from fruit waste, including phytochemicals, dietary fibers, and polysaccharides which can then be used as functional ingredients for long-term health benefits. Several novel extraction technologies like ultrasound-assisted extraction (UAE), pressurized liquid extraction (PLE), and supercritical fluid extraction (SFE) could provide an alternative approach for successful extraction of the valuable bioactives from the fruit waste for their utilization as nutraceuticals, therapeutics, and value-added products. Most of these waste-derived secondary metabolites comprise polyphenols, which have been reported to have anti-inflammatory, insulin resistance-treating, cardiovascular disease-maintaining, probiotics-enhancing, or even anti-microbial and anti-viral capabilities. This review summarizes the current knowledge of fruit waste by-products in pharmacological, biological, and probiotic applications and highlights several methods for identifying efficacious bioactive compounds from fruit wastes.

## Graphical Abstract

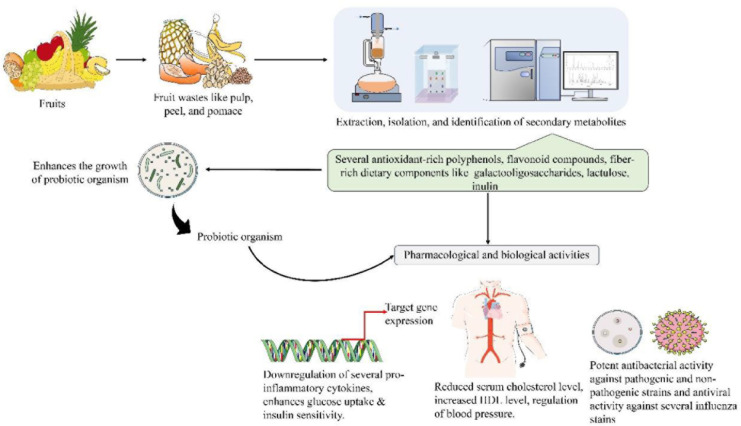

## Introduction

Food is a fundamental element necessary survival and life. Food waste is generated in different phases, i.e., during industrial manufacturing, processing, distribution, and agricultural production. Household activities contribute to about 42% of the total food waste, 39% by the food processing industries, 14% by the food service sector, and 5% during distribution. It was estimated that food waste may rise to 126 million tonnes by 2020 if improvements are not implemented (Mirabella et al., 2014; Baig et al., 2019). India, which has a population of more than 1.4 billion, generates more than 0.5 kg of organic waste per person per day (Paulraj et al., 2019). By separating high-value components such proteins, fibers, phytochemicals, flavor compounds, and polysaccharides, which can then be employed as functional ingredients and nutraceuticals, this prevention can be achieved (Baiano, 2014). There have been a number of initiatives in recent years to create strategies for therapeutically utilizing food (vegetable and fruit) waste. The agro-industrial waste is, however, utilized in huge quantities in the form of animal feed or fertilizers (Rudra et al., 2015).

Some recent reports showed that high-value products were developed by these types of fruit and agro-wastes and used in regular human lifestyles, such as (medicines, food items, and cosmetics) (Rudra et al., 2015; Kandemir et al., 2022). Researchers are looking for natural bioactive substances for treating and preventing several human diseases (Kandemir et al., 2022). These substances efficiently interact with biological molecules, including proteins, DNA, and others, to achieve the desired effects, which are then used to create natural therapeutics (Ajikumar et al., 2008). According to current research, consumers are becoming increasingly interested in food bioactives because of their potential to help people in various ways, such as preventing sickness and promoting good health. To achieve beneficial functional fruit products, it is essential to gather detailed information about the bio-actives (Kumar, 2015). Among these, nutraceuticals are therapeutic foods that significantly improve one’s health, increase immunity, and prevent and cure several diseases. Phytochemicals, on the other hand, have a specific role in showing positive effects on human health (Bansal and Priyadarsini, 2022). Currently, phytochemicals having potential cancer-preventive attributes are prioritized more (Kumar and Kumar, 2015). In recent years, there has been a growing trend in the food industries for the development of functional and nutraceutical products. Due to increasing consumer preference for “healthy” foods, this new category of food products has attracted much attention in the food industry. Hence, finding recent naturally occurring bioactive molecules that can be employed as nutraceuticals, functional food additives, or medicines has become of common interest to the pharmaceutical and food industries (Joana Gil-Chávez et al., 2013; Vilas-Boas et al., 2021). Fruit wastes provide a reliable source of secondary metabolites for the development of possible food additives, functional foods, preservatives, and nutraceuticals (Bhardwaj et al., 2022). Fruits and vegetable wastes represent the simplest form of functional foods because they are highly rich in several bioactive compounds. Fruits waste containing polyphenols and carotenoids showed antioxidant activity and reduce the risk of acquiring certain types of cancers (Day et al., 2009). The isolated bioactive molecules and by-products can be used to develop several functional foods in food processing industries and medicinal or pharmaceutical preparations (Baiano, 2014). Pomaces and other wastes from different fruits keep nutrients and bioactive substances, including phenolic acids, flavonoids, anthocyanins, and carotenoids, with a variety of biological functions (Das et al., 2021; Suri et al., 2022).

Fruit waste is becoming more appealing for research because these residues are a significant source of polyphenols (Lucarini et al., 2021). Fruit peel is the principal waste product in the food processing industries that use fruits as raw material, such as the manufacturing of fruit juices, jams, and dried fruits. Recently, researchers have become increasingly interested in exploring the antioxidant effects of fruit waste, such as peel and pomaces (Arias et al., 2022; Bello et al., 2023). Antioxidants can influence the expression of transcription factors involved in the immune response, reduce pro-inflammatory cytokine expression, and block crucial immune signaling pathways (Yahfoufi et al., 2018). The nuclear factor kappa B (NF-κB) family is one of the most significant signaling pathways that govern immunological responses and inflammation (Lawrence, 2009). The most abundant complex is p65/p50, which regulates gene expression of interleukin (IL)-1, IL-6, IL-8, inducible nitric oxide synthase (iNOS), IFN-γ, tumor necrosis factor (TNF)-α, and cyclooxygenase (COX)-2 (Modak and Bhattacharjee, 2022). Irregular activation of these pathways leads to several inflammatory diseases and even autoimmune diseases like rheumatoid arthritis (RA) and even cancer. Plasma-membrane-bound ligands, including toll-like receptor (TLR) and IL-1, can activate this pathway, resulting in the phosphorylation of IκB and its breakdown. As a result, NF-κB translocates into the nucleus and begins to upregulate transcription factor genes, which regulate cell inflammatory responses and survival (Modak and Bhattacharjee, 2022).

**FIGURE 1:**
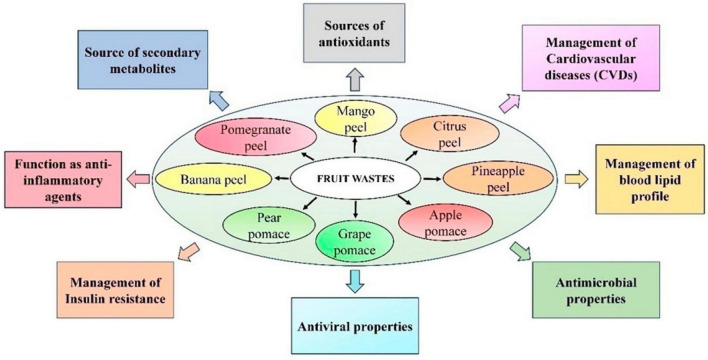
Pharmacological attributes of various edible fruit wastes. Figure generated using Microsoft PowerPoint 2021, 64-bit (Version 2302, Build 16130.20306).

Among the most common non-communicable diseases, cardiovascular disease (CVD) accounts for around 17.7 million deaths worldwide (WHO, 2016). India accounts for around one-fifth of these deaths worldwide (Kumar and Sinha, 2020). CVDs have wide variety of outcomes, including cerebrovascular disease, stroke, atherosclerosis, diabetes, coronary heart disease, obesity, and hypertension (Petrie et al., 2018). High blood cholesterol is also essential in the development of CVD (American Heart Association, 2012). Dietary patterns rich in lipids and cholesterol increase the risk of atherosclerosis, leading to CVD (Stocker and Keaney, 2004; Carson et al., 2020). Atherosclerosis indicates the accumulation of cholesterol within the arterial walls, thereby narrowing the arteries and forming atherosclerotic plaques (Brody, 1999). The disease manifestation of atherosclerosis is mainly due to endothelial damage (Caliceti et al., 2022). Currently, long-term pharmacological therapy is the most common strategy to control CVDs. However, most of these therapeutic approaches are inefficient for all patients and often elicit several side effects (Zhao et al., 2017). So, there is a growing interest in identifying alternative natural resources to combat the increasing incidence of CVD. In this context, natural compounds with potent antioxidant and free radical scavenging activities have drawn increased attention from the scientific community (Fu et al., 2011). Numerous studies support the direct relationship between a diet rich in vegetables and fruits and with low risk of CVDs (Figure 1; Cicero et al., 2017; Marracino et al., 2022).

Fruit waste extract also represents a novel strategy for combating harmful bacteria and viruses. Polyphenols are secondary metabolites generated from various portions of edible fruits wastes (apple, citrus, banana, pomegranate, grape, and pear) that include one or more phenolic groups (Gerardi et al., 2021; Ko et al., 2021; Zardo et al., 2021; Hasan et al., 2022). They not only have numerous human health benefits such as anti-diabetic, anti-cancer, antioxidant, and cardioprotective, but additionally, they possess anti-microbial-and antifungal properties (Guo et al., 2020; Saleem and Saeed, 2020; Budiati et al., 2022; Elbandrawy et al., 2022; Ko and Ku, 2022; Lee et al., 2022). Furthermore, there is an increasing prevalence of drug resistance to harmful bacteria, which is a severe threat to humanity. Thus, it is crucial to select the most appropriate antibiotics and employ them properly (Figure 1; Bains and Chawla, 2020). The potential of fruit wastes may be increased by using them as a base for the discovery of bioactive compounds, which could have significant positive effects on the food industry and medicinal fields.

## As a source of secondary metabolites, anti-oxidant, and anti-inflammatory agents

Apple pomace or peel is a by-product of the extraction process used to make apple juice and cider, and it is one of these sources that is considered as having substantial potential as a food ingredient (Kolodziejczyk et al., 2007). It is a solid mass that makes up to 30% of the fruit’s weight and comprises leftover peel, seed, stem, and pulp. Due to the presence of phenolics such as chlorogenic acid, quercetin glycosides, epicatechin, its dimer, phloridzin, and 3-hydroxyphloridzin, apple pomace has high antioxidant capabilities (Lu and Foo, 2000). According to several reports, apple pomace possesses good ferric-reducing ability power (FRAP) activity and 2,2′-diphenyl-1-picrylhydrazyl (DPPH)-scavenging move (Garcia-Montalvo et al., 2022; Llavata et al., 2022). Identification and quantification of significant phenolics by reverse phase-HPLC study show the presence of quercetin, phloretin, and phloridzin in apple pomace (Rana et al., 2015). In a recent survey polyphenolic composition was determined using UHPLC-DAD-ESI-MS and results showed that apple pomace composed of quercetin 3-O-arabinofuranoside (13%), quercetin-3-O-rhamnoside (23%), quercetin-3-O-galactoside (27%), and the dihydrochalcone phloretin-2-O-glucoside (14%) (Fernandes et al., 2019). Various novel extraction techniques like ultrasound-assisted extraction (UAE) ultra-turrax extraction (UTE), are used nowadays to isolate phenolics compounds like phloridzin from fruit wastes. Recent studies showed that the UAE method yields slightly higher phloridzin content (55.86–71.19 μg GAE/g of fresh apple pomace) compared to the UTE method (58.39–64.43 μg/g of fresh apple pomace) (Pollini et al., 2021). However, several other studies have reported slightly lower phloridzin content while extracting polyphenols using the UAE method from various apple pulps, ranging from 11.40 to 40.91 μg/g of fresh pulp (Li et al., 2019). Similarly, when utilizing the UTE method on freeze-dried apple pulps, the reported phloridzin content ranged from 39.9 to 77.0 μg/g of dry weight (Santarelli et al., 2020). The by-products from the processing of pear fruit, called pear pomace, remain rich in bioactive substances, carbohydrates, and fibers that can be used medicinally. According to a study of phenolic compounds in various pear sections, pulps have the lowest phenol concentration containing bioactive molecules, which are after that more abundant in skins and seeds (Kolniak-Ostek and Oszmiański, 2015). Because of this, pear pomace is a rich source of these bioactive components. According to HPLC studies, five phenolic acids (chlorogenic acid, gallic acid, ferulic acid, vanillic acid, and p-coumaric acid), two triterpene compounds (oleanolic acid and ursolic acid), one phenolic glucoside (arbutin), and three significant flavanols (catechin, epicatechin, and rutin) were all detected in pear pomace (Li et al., 2014). According to quantitative studies of the total phenols, triterpenes, and flavonoids content, all components discovered in the peel were 6–20 times more concentrated compared to the pulp (Li et al., 2014).

Several phenolic and flavonoid compounds, including nobiletin, tangerine, and coumaric acid, have been identified and quantified from dried orange peel using HPLC-DAD (Shu et al., 2020). According to Czech et al. (2021), the peel of all citrus fruits had substantially more phenolic components than the pulp and among citrus fruit peel, green grapefruit peel had the highest concentration of ascorbic acid (67.36 mg/100 g) and lemon had the lowest (7.83 mg/100 g). The same study reported that limes, lemons, and mandarins have more DPPH radical scavenging ability than their pulp counterparts (Czech et al., 2021). Lee et al. (2010) successfully extracted polymethoxyflavones (PMFs) like nobiletin and tangeretin from *Citrus depressa* Hayata peels by supercritical fluid extraction (SFE) technique. Their results showed that these PMFs’ yield increased significantly when 85% aqueous ethanol was used as a modifier with 9.1% supercritical CO_2_ (Lee et al., 2010; Chien et al., 2022).

Another food product is pomegranate and due to its multiple medicinal properties, pomegranates have been utilized as traditional medicine for decades. In this context, pomegranate peels come into consideration as the peel also contains a mixture of bioactive components, and the synergistic interaction of various components can result in a wide range of physiological actions (Mo et al., 2022). Phytochemical screening showed that pomegranate peels contain high amounts of flavonoids, tannins, alkaloids, carbohydrates, terpenoids, and possess antioxidant activity (Khokar et al., 2021). Studies found that pomegranate peel extracts have the highest soluble phenolic content and higher antioxidant activity than other wastes (Gulsunoglu et al., 2019). The soluble phenolics of pomegranate peel extract contained derivatives of ellagic acid, gallic acid, punicalagin, and cyaniding (Table 1). Punicalagin was shown to be the most hydrolyzable tannin in pomegranate husks, followed by gallic acid, catechin, epicatechin, and ellagic acid (Gulsunoglu et al., 2019). A recent study by Parisi et al. (2020) reported that ellagic acid (ellagitannins) and gallagic acid derivatives (gallagyl esters) are the primary components of pomegranate peel (Parisi et al., 2020). A recent study by García et al. (2021) have successfully extracted punicalagin (17 ± 3.6 mg/g DW) from pomegranate peel extract (PPE) using pressurized liquid extraction (PLE) technique under optimal conditions of 200°C temperature and 77% ethanol (García et al., 2021). The phenolic compound punicic acid was also extracted from pomegranate seed using the SFE technique at optimized temperature (60°C) and pressure (320 bars) (Natolino and Da Porto, 2019). Grape pomace is rich in the phenolic compounds such as resveratrol, anthocyanins (cyanidin, delphinidin, malvidin, and petunidin derivatives), flavonols (quercetin, laricitrin, syringetin, and myricetin glycosides), and flavan-3-ols (catechin/epicatechin and their procyanidin oligomers), as detected in LC-MS/MS analysis (Table 1; Parisi et al., 2020). Duba et al. (2015) have successfully extracted polyphenols from grape peels and seeds using the sub-critical water extraction (SBWE) technique in semi-continuous mode, yielding 44.3–77 and 44–124 mg/g from peels and seeds, respectively (Duba et al., 2015). Other novel extraction methods like UAE and pulsed electric fields were also used to extract anthocyanins and other phenolic compounds from grape peel and pomace (Ghafoor et al., 2011; Maza et al., 2019). Ashraf-Khorassani and Taylor (2004) have successfully removed more than 79% of catechin and epicatechin from grape seeds using SFE with 40% methanol-modified CO_2_ (Ashraf-Khorassani and Taylor, 2004). Around 29–40% of all pineapple waste is made up of pineapple peel and is abundant in epicatechins, catechins, ferulic acids, and gallic acid, all of which can be used as active antioxidant components (Jatav et al., 2022). This naturally occurring ferulic acid can be extracted using conventional and non-conventional methods. The Soxhlet extraction using methanol and petroleum ether as solvents showed the best results for the extraction of ferulic acid from the pineapple peel powder with a percentage yield of 90.5% mg (Madhumeena et al., 2021). The percent yield of pineapple peel extracts through solvent extraction is 82% mg compared to the product through SFE (79% mg) (Madhumeena et al., 2021). Lun et al. (2014) also found a higher yield of ferulic acid from the autoclaved pineapple wastes (3.65 mg/g) compared to the non-autoclaved pineapple wastes (0.64 mg/g) (Lun et al., 2014).

**TABLE 1:** Biological activities of various edible fruit waste and their secondary metabolites.

Fruit waste	Bioactives	Biological activity			
		Anti-inflammatory activity	Management of insulin resistance	Cardiovascular disease	Effect on blood lipid profile
Apple pomace	Epicatechin, quercetin glycosides, chlorogenic acid, phloridzin, and phloretin (Lu and Foo, 2000; Rana et al., 2015; Fernandes et al., 2019)	Modulation of NF-κB signaling pathway (Choy et al., 2019; You et al., 2022); inhibition of secretory phospholipase, COX-2 activity (Kim, 2015); suppression of NO production (Lee et al., 2020); downregulation of IL-1β, TNF-α, IL-6 (Lee et al., 2022)	Improved glucose and lipid by translocation of GLUT4 (Shen et al., 2017); enhanced glucose uptake and insulin sensitivity by activating PPAR-γ (Kumar et al., 2019); enhanced glucose absorption (Chen et al., 2022)	Can lower serum uric acid (SUA) levels by xanthine oxidase inhibition (Cicero et al., 2017); iNOS activity regulation in a human endothelium-derived cell line (Waldbauer et al., 2016)	*In vitro* studies confirmed the lowering of lipid accumulation by pre-adipocytes (Ko and Ku, 2022); 4 weeks of consumption can reduce the TC and LDL-cholesterol levels in humans (Ravn-Haren et al., 2013; Szabo et al., 2022).
Pear pomace	Arbutin, ursolic acid, epicatechin, catechin, rutin, vanillic acid, gallic acid, oleanolic acid, chlorogenic acid, ferulic acid, p-coumaric acid (Li et al., 2014)	LOX inhibitors (Lončarić et al., 2021); inhibition of NO, COX-2, iNOS production (You et al., 2022)	Antihyperglycemic activity in diabetic models (both alloxan and STZ induced) (Mechchate et al., 2021; Yasmin et al., 2021)	Protect against LPS-induced myocardial injury via the ER pathway (Zhang et al., 2019)	Hypolipidemic potentials in the dexamethasone-induced diabetic rat (Velmurugan and Bhargava, 2013) lowered TG, TC, LDL, and VLDL levels but increased the cardioprotective lipid HDL level (Velmurugan and Bhargava, 2013; Chang et al., 2017)
Orange peel	Coumaric acid, nobiletin, tangeretin, ascorbic acid (Matsuo et al., 2019; Shu et al., 2020; Czech et al., 2021); hesperidin (Samota et al., 2023)	Membrane stabilization activity, protection of protein denaturation (Malik et al., 2021); inhibition of iNOS, NO, and COX-2 (Rong et al., 2021); amelioration of acute ear inflammation (Padilla-Camberos et al., 2022)	Improve glucose tolerance (Nguyen-Ngo et al., 2020); increase insulin action by downregulating the MEK-ERK1/2 signaling cascade in hepatocytes (Guo et al., 2020); protects against β-cell loss in diabetic mice model (Kaneko et al., 2022)	Protective function against ISO-induced myocardial infarction in rats (Paul et al., 2017); can prevent cardiovascular disease by regulating the systolic and diastolic blood pressure in diabetic rats (Khan et al., 2021)	Can reduce the TG and TC levels, and prevent LDL oxidation, thereby inhibiting atherosclerosis progression (Benavente-Garcia and Castillo, 2008; Feng et al., 2020)
Pomegranate peel	Gallic acid, ellagic acid, cyanidin, punicalagin, catechin (Zahin et al., 2010; Gulsunoglu et al., 2019; Parisi et al., 2020)	Inhibition of COX-2, IL-6, TNF-α, and iNOS (Du et al., 2019); downregulation of MMPs and NF-κB pathway (Calabriso et al., 2022)	Decreased hepatic gluconeogenesis and enhanced glycogenesis (Jin et al., 2020); protection of pancreatic β-cells in STZ-induced diabetic rat model (Abdulhadi et al., 2022; Elbandrawy et al., 2022)	Cardioprotective effect against Dox-induced cardiotoxicity in rats via the reduction in GSH, LDH, and creatine kinase-MB (Hassanpour Fard et al., 2011); prevents the onset of atherosclerosis progression by increasing expression of hepatocyte paraoxonase 1 expression in a dose-dependent manner (Khateeb et al., 2010); decrease advanced atherosclerotic progression and reduce plaque necrosis in Apoe^–/–^ mice (Manickam et al., 2022)	Can significantly reduce TG, TC, LDL-cholesterol, and lipid peroxidation in hypercholesterolemic rats (Ismail et al., 2012; Salama et al., 2021)
Grape pomace	Resveratrol, quercetin, laricitrin, syringetin, delphinidin, malvidin (Parisi et al., 2020; Sabra et al., 2021)	Downregulation of TNF-α, NF-κB, IFN-γ, IL-1α, IL-1β, and IL-10 (Pistol et al., 2019); inhibition of COX-2 enzyme (Lončarić et al., 2021)	Attenuated insulin resistance on animal model (Lanzi et al., 2016); improved fasting insulinemia (Martínez-Maqueda et al., 2018); improved insulin sensitivity and reduced ectopic fat deposition (Daniel et al., 2021)	Can significantly attenuate atherosclerosis development in SR-B1 KO/ApoER61h/h mice (Rivera et al., 2019), Inhibit LDL oxidation and thereby can prevent the onset of atherosclerosis development (Pérez-Jiménez and Saura-Calixto, 2008); *in vivo* studies showed cardioprotective effects by measuring creatine kinase levels and by ECG monitoring (Pop et al., 2018)	Can significantly reduce blood TG and VLDL level, with a slight increment of HDL level, and no alteration of TC level in high-fat diet-induced rat model (Smith et al., 2017; Martínez-Meza et al., 2022)
Banana peel	Gallic acid, catechin, anthocyanins, kaempferol, isoquercitrin (Zaini et al., 2022)	Act as LOX inhibitors (Lončarić et al., 2021); inhibition of NF-κB signaling (Eddie-Amadi et al., 2022); Suppression of IL-6, TNF-α, normalization of activated T-cell (Hong et al., 2023)	Enhanced glucose uptake in myoblast cells (Arun et al., 2017); upregulation of PI3K, AKT, IRS-1 expression and improved insulin resistance in STZ-induced diabetic mice model (Wang et al., 2022)	Can ameliorate the cardiac function by decreasing the elevated serum creatine kinase-MB, aspartate aminotransferase, and lactate dehydrogenase activities in nicotinamide/streptozotocin-induced diabetic rats (Ahmed et al., 2021)	Can significantly reduce the TC, TG, LDL-cholesterol, and VLDL-cholesterol levels in nicotinamide/streptozotocin-induced diabetic rats (Ahmed et al., 2021; Indriawati and Atiyah, 2022)
Pineapple peel	Gallic acid, catechins, epicatechins, and ferulic acids (Jatav et al., 2022)	Reduction of inflammatory cell counts and TNF-α, IL-6, levels (Ajayi et al., 2022)	Prevents insulin resistance in HepG2 cells (Hu et al., 2019)	Can significantly decrease the atherogenic and coronary risk index in HFD-fed rats (Ajayi et al., 2021)	Can significantly improve HDL level and significantly reversed the elevated LDL in HFD-fed rats (Ajayi et al., 2021)
Mango peel	Lupeol/Fagarsterol (Han and Bakovic, 2015); mangiferin (Mistry et al., 2023)	Decreased NO production (Huang et al., 2018)	Anti-diabetic property in STZ-induced diabetic rat model (Gondi et al., 2015)	Can decrease the activity of cardiac enzymes like LDH, AST, ALT, and ALP, thereby suggesting the cardioprotective effects in high-cholesterol diet-fed rats (Sudhahar et al., 2007); showed significant cardioprotective effects against vascular damage caused by LDL oxidation in obese females (Arshad et al., 2021)	Can reduce the TC and TG levels in high-cholesterol diet-fed rats (Sudhahar et al., 2007); showed significant reduction of TG, LDL, and VLDL-cholesterol in the alloxan-induced type I diabetic rat (Mistry et al., 2023)

**FIGURE 2:**
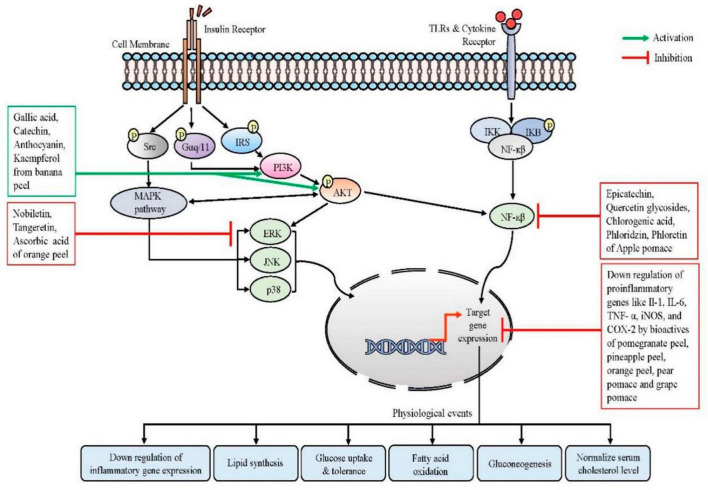
Modulation of intracellular signaling cascades by different edible fruit wastes, their phyto-components, and their physiological events during homeostasis. Binding with insulin receptors, insulin signals stimulate MAPK kinase pathways and activate PI3K, which in turn activates AKT. Phyto-components of banana peel can activate this pathway. AKT also interacts with NF-κβ pathways. Activation of the TLR initiates an inflammatory cascade that leads to the activation of NF-κβ and the production of pro-inflammatory cytokines. Bioactives from several fruit wastes can modulate the NF-κβ signaling pathways, which results in the downregulation of inflammatory biomarkers. Similarly, the MAPK pathway and AKT eventually control the metabolic gene expression, which ultimately restores the normal glucose uptake, promoting fatty acid oxidation and controlling blood cholesterol levels. Src, proto-oncogene tyrosine-protein kinase; TLR, toll-like receptors; Gαq/11, a family of heterotrimeric G protein alpha subunits; PI3K, phosphoinositide 3-kinase; IRS, insulin receptor substrate; AKT, serine-threonine protein kinase; MAPK, mitogen-activated protein kinase; JNK, c-Jun N-terminal kinases; ERK, extracellular signal-regulated kinase; NF-κβ, nuclear factor kappa beta; IKB, inhibitor of NF-κβ; IKK, IKB kinase. Figure generated using Microsoft PowerPoint 2021, 64-bit (Version 2302, Build 16130.20306).

Due to their synergistic and additive effects, the complex blend of phytochemical components present in fruit-peel extracts is more efficient for preventing inflammation than their components. Polyphenols have shown significant effectiveness in controlling this pathway at various points. The flavonoids like quercetin, kaempferol, myricetin, and apigenin have been shown to inhibit serine/threonine protein kinases (PIK3/AKT) in an antagonistic manner. They can modulate transcription factors such as NF-κB (Choy et al., 2019). Gallic acid, resveratrol, and quercetin can inhibit COX-2 enzymes, whereas lipoxygenase (LOX) inhibitors include kaempferol, ferulic acid, benzoic acid, quercetin, caffeic acid, and catechin (Figure 2; Lončarić et al., 2021). *In vitro* study demonstrated that the 70% ethanolic apple peel-extract can substantially inhibit secretory phospholipase, COX-1, COX-2, and lipoxygenase activity by up to 53.5 ± 2.3, 13.4 ± 1.8, 64.8 ± 5.4, and 44.4 ± 4.5 (percentage ± SD), respectively (Kim, 2015). Another culture-based study demonstrated that the ethanolic apple peel extract can suppress nitric oxide (NO) production up to 25% at a dose of 500 μg/ml in lipopolysaccharide (LPS)-induced Raw 264.7 macrophage cell line (Lee et al., 2020). This study further confirmed that apple peel extract effectively inhibited LPS-induced production of pro-inflammatory iNOS and COX-2, as well as phosphorylation of NF-κB subunit p65 and further real-time PCR confirmed the expression of pro-inflammatory biomarkers like prostaglandin E synthase 2 (PTGES2), monocyte chemoattractant protein-1 (MCP-1), IL-6, and IL-1β were also significantly downregulated in an in a dose-dependent way (Lee et al., 2020). A more recent study by the same research group revealed that isoquercitrin was the main bioactive extracted from green ball apple peel extract (Lee et al., 2022). Isoquercitrin can inhibit NF-κB, COX-2, iNOS, and p65 protein expressions along with proinflammatory markers like TNF-α, IL-6, and IL-1β in a concentration-dependent fashion in LPS-treated Raw 264.7 macrophage cell line (Figure 2; Lee et al., 2022). A recent study reported that the ethanolic extract of pear pomace can also enhance LPS-induced inflammation in RAW 264.7 cells by decreasing NF-κB expression and NO production (You et al., 2022). Another work by Martins et al. (2017) reported that extracts of grape pomace containing phenolic compounds (in the concentration of 100 μg/ml) can decrease COX-2, prostaglandin (PGE)-2, and IL-8 production in Caco-2 cells pre-treated with leukocyte IL-1. Further HPLC analysis indicated that this extract had a high amount of flavonoids, including catechin, gallic acid, and epicatechin, which may help to modulate PGE-2 and IL-8 release (Martins et al., 2017).

According to Malik et al. (2021), *Citrus nobilis* peel methanolic extract can protect RBC membrane stabilization by up to 89.67% and can protect the protein denaturation by up to 87.57% at a dose of 200 mg/ml concentration. Most biological proteins lose the three-dimensional structures and their physical function due to heat exposure, which triggers several hypersensitive immune responses and forms chronic inflammatory arthritis (Modak et al., 2021). Due to the presence of sinensetin, nobiletin, and other bioactive components, *C. nobilis* peel methanolic extract may restore protein integrity by preventing membrane protein rupture by widening the surface-volume ratio and by reducing the release of inflammatory mediators (Malik et al., 2021; Modak et al., 2021). Padilla-Camberos et al. (2022) reported that topical use of a natural essential oil mix of sweet orange peel, allspice, and cumin seeds could ameliorate ear inflammation by 66.67% when compared to the negative control group, and that may be due to the synergistic or additive effect (Padilla-Camberos et al., 2022). Further studies revealed that citrus peel flavonoid nobiletin can exert anti-inflammatory effects by reducing the expression of iNOS, COX-2, and NO production from both the protein and gene level in LPS-treated RAW macrophage 264.7 cell line (Figure 2; Rong et al., 2021).

Ellagic acid, one of the primary bioactive of pomegranate peel extract, can inhibit the expression of pro-inflammatory cytokines like IL-6, TNF-α and IL-1, as well as down-regulate the COX-2 and iNOS expression in macrophage Raw 264.7 cell line (Du et al., 2019). This study further reported that polyphenols from pomegranate peel can inhibit the mRNA and protein production of TLR4 and could inhibit p65 nuclear translocation by inhibiting NF-κB activation by preventing LPS-induced phosphorylation, ubiquitination, and degradation of IκB (Figure 2; Du et al., 2019). Another recent study by Calabriso et al. (2022) showed the anti-inflammatory effects of red grape pomace on LPS and TNF-induced inflammation in Caco-2 cells and HMEC-1 cells. According to their study, they found that treatment with grape pomace reduced the levels of IL-6 and MCP-1 (monocyte chemoattractant protein-1) as well as the production of MMP-9 and MMP-2 in a dose dependant manner in HMEC-1 cell line. Furthermore, they noted that treatment with the red grape pomace also downregulated the NF-κB pathway which ultimately inhibited the gene expression of several pro-inflammatory markers like COX-2, TNF-α, IL-1β, and macrophage colony-stimulating factor (M-CSF) (Calabriso et al., 2022). The peels of pineapple and banana has been reported to possess several phenolic compounds like gallic acid, catechin, epicatechin, anthocyanins, kaempferol, and isoquercitrin (Jatav et al., 2022; Zaini et al., 2022). These phenolic compounds and flavonoids can be successfully extracted using UAE method at optimal extraction conditions. Using UAE technique at optimal parameters (30°C, 5 min, 150 W), 1 g of *Musa cavendish* peel can yield up to 23.49 mg of phenolic compounds, 13.11 mg of proanthocyanidins, and 39.46 mg of flavonoids (Vu et al., 2017). The presence of such phenolic-rich constituents in banana peel extract has been reported to exert an anti-inflammatory role by inhibiting the NF-κB pathway (Eddie-Amadi et al., 2022). Another recent study found that banana peel can inhibit the expression of IL-6 production in the LPS-stimulated RAW264.7 cells (Table 1). According to the *in vivo* study in mice model, the banana peel extract has been reported to lower the elevated serum TNF-α, IL-6 levels, and also normalized the activated T-cell population (Hong et al., 2023). Parisi et al. (2020) reported the anti-inflammatory activity of herbal formulation comprising of propolis, pomegranate peel, and aglianico grape pomace extracts (4:1:1) in collagen-induced arthritis (CIA) model (Parisi et al., 2020). According to their study, early treatment with the herbal dose (150 mg/kg body weight) can significantly ameliorated the paw swelling and also reduced the number of affected paws by 60% when compared with negative control mice. Furthermore, the downregulation of IL-17 and IL-1β cytokines at protein levels in the treatment group, also confirmed the anti-rheumatoid activity of the herbal formulation (Table 1; Parisi et al., 2020).

## Management of insulin resistance

Insulin resistance (IR) is one of the major symptoms of a number of human diseases, including CVD, obesity, type 2 diabetes, polycystic ovary syndrome, metabolic dysfunction-associated fatty liver disease and others (James et al., 2021; Li et al., 2022). Current understanding states that adipose tissue could have an essential role in the development of IR through the generation of lipids and other circulating substances that induce IR, as well as in the insulin-mediated regulation of glucose metabolism in other organs (Li et al., 2022). In healthy individuals, the transport of the glucose transporter solute carrier family 2 protein facilitated GLUT-4 to the cell surface and promotes cellular glucose absorption from the circulation into tissues (James et al., 2021; Li et al., 2022). This procedure eliminates high postprandial glucose from the blood, however, a habitually high glycemic index diet results in excessive insulin production and increased binding to the insulin receptor, leading to fatty acid synthesis in addition to the typical glucose uptake. The PI3K and the MAP kinase pathway are the two primary pathways that are activated as a result of all of these activities (Figure 2; Li et al., 2022). Any change in these two pathways hindered glucose re-uptake in both the adipose tissues as well as the skeletal muscle tissues, contributing to the phenomenon of IR. Several studies have shown that the high anti-oxidant activity of fruit waste can help to improve and manage IR and type 2 diabetes through their synergic effects. In differentiated adipocytes, phloretin and phloridzin, two of the primary active components of apple peel, stimulate peroxisome proliferator-activated receptor (PPAR)-γ and inhibit cyclin-dependent kinase (Cdk)-5 to enhance glucose uptake and insulin sensitivity (Kumar et al., 2019). Phloretin reduces IR *in vitro* and improves the glucose and lipid metabolism in streptozotocin-induced diabetes rats by translocation of GLUT4 (Table 1; Shen et al., 2017). According to Chen et al. (2022), phloridzin derivatives are a potential therapy for type 2 diabetes patients who have IR. In untreated C2C12 myotubes, docosahexaenoic acid-acylated phloridzin, a new polyphenol fatty acid ester derivative, enhanced glucose absorption and mitochondrial function via enhancing AMPK activity (Chen et al., 2022). A novel formulation that has the potential to be an important replacement for conventional medications is validated by the synergistic combination of pear pomace, another rich source of bioactive (Li et al., 2014). The anti-diabetic formulation containing flavonoids such as catechin, epicatechin, and rutin showed substantial anti-hyperglycemic activity at 10 mg/kg body weight dose in the alloxan-induced diabetic mice model (Mechchate et al., 2021). Ferulic acid, another important polyphenol from pear pomace also improved hyperglycemia significantly in STZ-nicotinamide-induced diabetic rat model (Yasmin et al., 2021). Nobiletin and tangeretin were important flavonoids identified from the orange peel that also possess anti-diabetic activity (Table 1). Nguyen-Ngo et al. (2020), reported that nobiletin treatment can substantially boost TNF-related glucose uptake in human skeletal muscle. Additionally, it was observed to downregulate the expression of pro-inflammatory markers at both the mRNA and protein levels in human placenta and visceral adipose tissue. Furthermore, the study conducted on gestational diabetes mellitus mice model revealed that, nobiletin at 50 mg/kg body weight dose can significantly improve glucose tolerance and can inhibit Akt activation in the placenta (Nguyen-Ngo et al., 2020). The intragastric injection of tangeretin at a dose of 50 mg/kg body weight was found to improve glucose homeostasis and hepatic insulin sensitivity in diabetic mice with genetically altered leptin receptors. The results suggest that tangeretin may have the ability to increase insulin action by downregulating the MEK-ERK1/2 pathways in hepatocytes (Figure 2; Guo et al., 2020). According to Kaneko et al. (2022), the subcutaneous administration of nobiletin led to a significant reduction in blood glucose levels in non-fasting condition and during an OGT test in male diabetic db/db mice model. Indeed, the study further confirmed that continuous administration of nobiletin to db/db mice resulted in a larger β-cell mass and higher insulin content compared to the vehicle control group. These findings suggest that suggesting nobiletin not only improves IR but also offers protection against β-cell loss in the type 2 diabetic db/db mice model (Kaneko et al., 2022). As per other studies, ellagic acid, cyanidin, and punicalagin are potentially promising supplements for lowering diabetes-related insulitis. Treatment with ellagic acid 50 mg/kg can enhance the islets of Langerhans in STZ-induced diabetic rats by downregulating TNF-α and IL-6-induced systematic inflammation (Elbandrawy et al., 2022). Jin et al. (2020) reported that punicalagin treatments activated the PI3K/AKT signaling pathway, which decreased hepatic gluconeogenesis and enhanced glycogenesis, both in glucosamine-induced HepG2 cell line and STZ-induced mice model (Figure 2). In another study, it was found that punicalagin exhibited protective effects on β-cells in the pancreas of STZ-induced diabetic Wistar rats. Additionally, punicalagin was observed to reduce oxidative stress levels and enhance antioxidant levels in diabetic rat model (Abdulhadi et al., 2022). Punicalagin’s effects on the liver and kidney, resulting in hypoglycemic and anti-inflammatory effects, were primarily mediated by a decrease in gluconeogenesis and an increase in glycogenesis, as well as by activating the PI3K/AKT signaling pathway, controlling the inflammatory signaling network and by regulating gut microbiota homeostasis (Jin et al., 2020; Cao et al., 2021). Cyanidin, a different phenolic compound from pomegranate peel, can also ameliorate metabolic IR. A recent study by Geng et al. (2022) reported that cyanidin-3-O-glucoside supplementation improves metabolic IR in STZ-induced mice models by downregulating inflammatory cytokines and TLR4/NF-κB inhibitor alpha (IκBα) activation and restoring the suppressed AKTt/eNOS signaling pathway (Figure 2). Such research continues to be beneficial in revealing a novel alternative method for treating IR in diabetic complications, which has a lot of potential in the future that involves turning fruit waste into a distinctive source of antioxidant-rich by-products.

## Management of cardiovascular diseases and blood lipid profile

Polyphenols extracted from apple pomace were known to exhibit cardiovascular protective effects by lowering serum uric acid (SUA) levels (by xanthine oxidase inhibition) and improving endothelial reactivity (ER) (Cicero et al., 2017). The triterpenic acids from methanol/water extracts of apple pomace can modulate the eNOS (endothelial nitric oxide synthase) activity in a cell line derived from human endothelium (Table 1; Waldbauer et al., 2016). Apple skin contains phloridzin, chlorogenic acid, and quercetin, which have the potential to regulate glucose absorption and can reduce postprandial glycemia (blood glucose levels after a meal) and insulinemia (insulin levels in the blood) (Makarova et al., 2015; Cicero et al., 2017). Various *in vitro* studies also confirmed the anti-obesity properties of apple peel extract by lowering lipid accumulation by pre-adipocytes (Ko and Ku, 2022). In healthy human volunteers, a daily uptake of apple, apple pomace, or apple juice for a duration of 4 weeks has been shown to lead to a reduction in both total cholesterol (TC) and LDL-cholesterol levels (Ravn-Haren et al., 2013). Phloridzin, chlorogenic acid, and catechin found in apple pomace have the potential to lower serum triglycerides (TG) and LDL-cholesterol levels while also helping in regulating glycemia (blood sugar levels) (Table 1; Szabo et al., 2022).

The biomolecules obtained from pear and its extracts has also many cardioprotective functions. The extracts of *Pyrus*, commonly known as Himalayan pear have rich content of arbutin, catechin, and chlorogenic acid and were known to inhibit the COX-2 activities and reduce IL-6 and TNF-α expression in LPS-stimulated RAW macrophages (Figure 2; Om et al., 2022). The arbutin treatment could also protect against LPS-induced myocardial injury via ER pathway in an *in vivo* model system through the modulation of TNF-α and IL-6 levels (Zhang et al., 2019). Pear and pear by-product extracts also showed hypoglycemic (blood-sugar lowering) and hypolipidemic (cholesterol lowering) potentials in the dexamethasone-induced diabetic rat (Velmurugan and Bhargava, 2013). This extract not only lowered TG, TC, LDL, and VLDL levels but also increased the cardioprotective lipid HDL level (Velmurugan and Bhargava, 2013). Chang et al. (2017) found out that the pear insoluble dietary fiber (IDF) can decrease the LDL-cholesterol and TC levels in high-fat-induced rats (Table 1; Chang et al., 2017). Therefore, the pear and its by-product extracts have the potency to prevent the formation of CVDs like atherosclerosis and coronary heart disease.

The extracts obtained from both the peel and pulp of the wild orange, *Citrus macroptera*, has been found to have cardioprotective effects. These extracts showed notable protective capabilities against isoproterenol (ISO)-induced myocardial infarction (MI) in rats (Paul et al., 2017). Citrus fruits’ peel, pulp, and seeds are rich in flavanone glycosides, with hesperidin being one of the prominent compounds in high amounts (Samota et al., 2023). Hesperidin (at a dose of 100 mg/kg) was found to control blood glucose levels effectively and showed cardioprotective functions in isoproterenol-induced MI in STZ-nicotinamide-induced diabetic rats (Kakadiya et al., 2010). Hesperidin also has blood pressure-reducing effects, and studies have confirmed its ability to prevent and treat CVDs by regulating both systolic and diastolic blood pressure in diabetic rats (Table 1; Khan et al., 2021). Various flavanone derivatives, such as hesperidin and naringin, in the juices of Citrus fruits has been verified to exhibit cholesterol-lowering activity in human subjects. The essential oils from the lemon and citrus peel can also reduce triglycerides and TC levels, and prevent LDL oxidation, thereby inhibiting atherosclerosis progression (Figure 2; Benavente-Garcia and Castillo, 2008; Feng et al., 2020).

Pomegranate, *Punica* sp.’s peels, and juice have various therapeutic potentials related to cardiovascular complications. Almost 92% of the antioxidant property of the fruit is mainly due to the presence of multiple flavonoids (such as anthocyanins and catechins) and tannins (such as gallic acid, ellagic acid, punicalin, and punicalagin) concentrated in the peels of pomegranate (Zahin et al., 2010). The hydroethanolic extract obtained from pomegranate peel, rich in polyphenols (such as punicalagin), showed a significant reduction of plaque necrosis and advanced atherosclerotic progression in Apoe^–/–^ mice *via* the suppression of MerTK cleavage and elevation of lesional macrophage efferocytosis (Table 1; Manickam et al., 2022). The water extract derived from pomegranate aril, rind, and seeds has been found to possess a cardioprotective effect against doxorubicin (Dox)-induced cardiotoxicity in rats via the reduction in glutathione, lactate dehydrogenase (LDH), and creatine kinase-MB (Hassanpour Fard et al., 2011). The pomegranate peel extract, owing to its rich antioxidant content, can enhance the hepatocyte paraoxonase one expression in a dose-dependent manner, thereby preventing the onset of atherosclerosis progression (Khateeb et al., 2010). Another study in hypercholesterolemic mice showed elevated eNOS expression and inhibition of atherosclerosis progression by uptake of punicalagin-enriched pomegranate juice (De Nigris et al., 2007). Studies in human subjects also suggest the importance of daily uptake of pomegranate juice as it can attenuate atherosclerosis and prevents hypertension (Wang et al., 2018). The long-term consumption of pomegranate peel powder can significantly reduce TC, TG, LDL-cholesterol levels, and lipid peroxidation in hypercholesterolemic rats (Figure 2; Ismail et al., 2012; Salama et al., 2021).

The bioactives obtained from grape extract (*Vitis* sp.) also possess many cardioprotective functions. Bioactive molecules like resveratrol and quercetin have been effective as anti-hypertensive and treatment of cardiac damage (Theodotou et al., 2017; Sabra et al., 2021). Grape pomace (GP) is a by-product of the winery industry that contains a high concentration of polyphenols and dietary fiber (Taladrid et al., 2022). Studies have suggested the *in vivo* cardioprotective effects of the pomace extract by measuring creatine kinase levels and ECG monitoring (Pop et al., 2018). Recent studies have demonstrated that red wine grape pomace can significantly attenuate atherosclerosis progression in mice with SR-B1 KO/ApoER61h/h (Rivera et al., 2019). The polyphenolics from grape extracts inhibit LDL oxidation and thereby can prevent the onset of atherosclerosis development (Pérez-Jiménez and Saura-Calixto, 2008). Grape pomace can also regulate blood lipid profile. Studies on the high-fat diet model (AIN-93G-induced) for ten weeks showed that with the increasing concentration of grape pomace, there was a noteworthy reduction in blood TG and VLDL level, with a slight increment of HDL level and no alteration of TC level (Table 1; Smith et al., 2017).

Lupeol (also known as Fagarsterol) is a naturally occurring pentacyclic triterpene predominantly found in high concentrations in mango peels (Han and Bakovic, 2015). Lupeol can be extracted from the mango peel using the UAE method as it enhances the extraction efficiency by shortening time and increasing yield (Ruiz-Montañez et al., 2014). Due to these pentacyclic triterpenoids, mango peel may possess cardioprotective functions. Studies in high-cholesterol-fed rats found that consumption of lupeol and its derivatives can reduce the TC triglyceride, and decrease the activity of cardiac enzymes like LDH, aspartate aminotransferase (AST), alanine aminotransferase (ALT), and alkaline phosphatase (ALP), thereby suggesting the cardioprotective effects of triterpenoids (Sudhahar et al., 2007). Mango peel powder has shown significant cardioprotective effects in obese females by protecting against vascular damage caused by LDL oxidation (Arshad et al., 2021). Both mango peel extract and mangiferin have been found to produce significant reductions in TG, LDL, and VLDL-cholesterol in the alloxan-induced type I diabetic rat (Table 1; Mistry et al., 2023).

## Antimicrobial and antiviral properties of fruit waste products

Apple pomace contains flavonoids, phenolic acids, carotenoids, and anthocyanins that have various anti-microbial and anti-viral activities (Kuznetsova et al., 2017; Barreira et al., 2019; Zardo et al., 2021). Polyphenolic bioactives from apple pomace showed antimicrobial activity against *Paenibacillus* larva (American foulbrood in honeybees) (Giménez-Martínez et al., 2020). Lipophilic compounds from different species of *Malus* (apple pomace) consist of saturated, unsaturated, and polyunsaturated fatty acids, along with polyphenols, phytosterols, and four homologs of tocopherol were found to exhibit inhibitory effects on the growth of a few Gram-positive and Gram-negative bacterial cultures such as *Escherichia coli, Pseudomonas aeruginosa*, *Enterococcus faecalis*, *Streptococcus pyogenes, Bacillus cereus*, *Micrococcus luteus*, *Bacillus subtilis*, and *Staphylococcus aureus* (Table 2; Alberto et al., 2006; Fratianni et al., 2011; Zhang et al., 2016; Kuznetsova et al., 2017; Radenkovs et al., 2018; Zardo et al., 2021). Phenolic compounds isolated from different extraction solvent varies in antimicrobial activities (Zhang et al., 2016; Zardo et al., 2021; Santos et al., 2022). Bioactive compounds such as phloridzin, phloretin, epicatechin, floridzine, procyanidin B2, chlorogenic acid, and quercetin were found to possess significant anti-bacterial activities against *S. aureus* and *E. coli* (Zhang et al., 2016; Zardo et al., 2021). Hydro-ethanolic extract of apple pomace also showed antibacterial activity against Gram-negative bacteria *Propionibacterium acnes* and *Proteus mirabilis* with minimal inhibition concentration (MIC) of 2.5 and 10 mg/ml, respectively (Table 2; Arraibi et al., 2021). However, in another study, it was shown that volatile components from apple pomace have anti-bacterial potential against *S. aureus*, *Salmonella typhimurium*, and *E. coli* with MIC of >12.5 mg/ml for the tested bacterial strains (Carpes et al., 2021).

**TABLE 2:** Antibacterial activity of different edible fruit wastes and their secondary metabolites.

Waste part of fruits	Bioactive compounds	Activity	References
Apple pomace	Phloridzin, phloretin, quercetin, chlorogenic acid, floridzine, procyanidin B2, and epicatechin	Against *P. aeruginosa*, *E. coli*, *E. faecalis*, *B. cereus*, *M. luteus*, *B. subtilis, S. pyogenes*, and *S. aureus*	Alberto et al., 2006; Fratianni et al., 2011; Zhang et al., 2016; Kuznetsova et al., 2017; Radenkovs et al., 2018; Zardo et al., 2021
Hydro-ethanolic extract of apple pomace	Polyphenolic bioactive compounds	Against *P. acnes* and *P. mirabilis*	Arraibi et al., 2021
Grape shells	Polyphenols	Against *S. aureus* and *L. monocytogenes*	De-Souza et al., 2014; Xu et al., 2016
Hydromethanolic extract of skin/seed of the grape	Flavonoid, anthocyanin, and proanthocyanidins	Against *E. faecalis, L. monocytogenes, L. innocua, E. coli, K. pneumonia, M. morganii*, and *P. aeruginosa*	Peixoto et al., 2018; Gerardi et al., 2021; Gómez-Mejía et al., 2021
Methanolic extract of pomegranate peel	Flavonoids, anthocyanins, phenolics, alkaloids, and tannins	Against *S. aureus, B. megaterium, L. monocytogenes, B. cereus, B. subtilis, P. aeruginosa, K. pneumonia, E. coli*, and *S. typhi*	Hasan et al., 2018; Abou El-Nour, 2019; Belgacem et al., 2020; Alsubhi et al., 2022
Ethyl acetate crude extract of pomegranate peel	Phenolics and flavonoids	Against *X. gardneri, P. carotovorum*, and *R. solanacearum*	Khaleel et al., 2018
*Citrus* spp. peel	γ-Terpinene, narirutin, limonene, naringin, hesperetin-7-O-rutinoside, linalool, naringenin, quinic acid, linalyl acetate, sakuranetin, and datiscetin-3-O-rutinoside	Against *E. coli*, *Bacillus* spp., *L. monocytogenes*, *E. faecalis, B. cereus*, *S. aureus*, and *S. typhimurium*	Oikeh et al., 2020; Shehata et al., 2021; Hasan et al., 2022; Cebi and Erarslan, 2023; Meryem et al., 2023; Saleem et al., 2023
Pear peels and pulp	Chlorogenic acid, arbutin, malaxinic acid, oleanolic acid, rutin, ursolic acid, epicatechin, and procyanidin B2	Antibacterial effects	Li et al., 2014; Hussain et al., 2022
Ethanolic/aqueous/ethyl acetate extract of banana peel	Malic acid, β-sitosterol, succinic acid, and palmitic acid	*S. typhimurium*, *B. cereus*, *S. aureus*, *L. monocytogenes, K. pneumoniae, P. vulgaris, S. pyogenes*, and *E. coli*	Saleem and Saeed, 2020; Maryati et al., 2021; Hanafy et al., 2021; Hikal et al., 2022; Anandhi and Rajeshkumar, 2023

**FIGURE 3:**
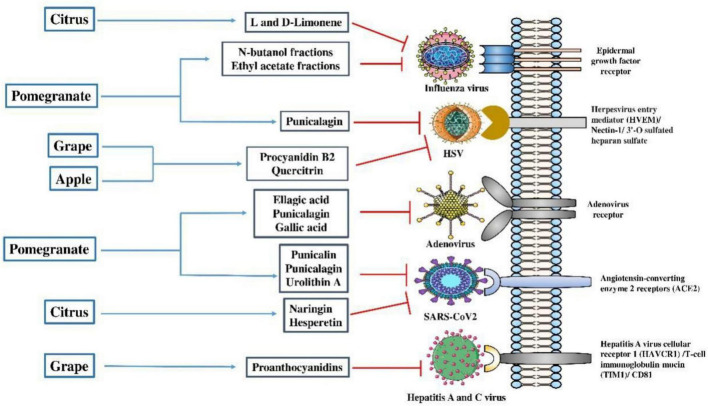
Mode of action of antiviral properties of secondary metabolites extracted from edible fruit wastes. Secondary metabolites like procyanidin B2 and quercitrin extracted from apple and grape wastes can inhibit the entry and replication of herpesvirus and hepatitis A and C virus. L and D limonene, naringin, and hesperetin extracted from wastes of citrus fruit showed anti-influenza and anti-SARS-CoV-2 virus action. Antiviral effects of ellagic acid, punicalagin, and gallic acid from pomegranate peel extracts were found against adenoviruses. SARS-CoV-2, severe acute respiratory syndrome coronavirus 2; HSV, herpes simplex virus. Parts of the figure were drawn using pictures from Servier Medical Art and generated using Microsoft PowerPoint 2021, 64-bit (Version 2302, Build 16130.20306).

*In vitro* studies reported that polyphenols from the methanolic extract of apple pomace can inhibit both type 1 and 2 of herpes simplex virus (HSV) viral replication at the concentration of 1,200 μg/ml (Table 3; Suárez et al., 2010). Phytochemicals such as quercitrin and procyanidin B2 were the most important compounds to have a role in the inhibition of HSV viruses by inactivating extracellular virions or inhibiting early viral replication events (Alvarez et al., 2012; Figure 3).

**TABLE 3:** Anti-viral activity of different edible fruit wastes and their secondary metabolites.

Waste part of fruits	Bioactive compounds	Activity	References
Methanolic extract of apple pomace	Procyanidin B2 and flavonol quercitrin	Both type 1 and 2 HSV	Suárez et al., 2010; Alvarez et al., 2012
Red grape seed extract	Proanthocyanidins	human norovirus surrogates, HSV-1, hepatitis C virus, HAV, porcine reproductive and respiratory syndrome virus (PRRSV), DENV	Joshi et al., 2015; Pasqua et al., 2016; Zhang et al., 2018; Oh et al., 2022; Pascual et al., 2022; Chen et al., 2023
Pomegranate peel n-butanol and ethyl acetate extracts	Gallotannins, tannins, and ellagitannins	Influenza A virus	Moradi et al., 2019
Pomegranate peel	Punicalagin	HSV-1	Houston et al., 2017
Pomegranate peel ethanolic extract	Ellagic acid, and punicalagin	Adenovirus	Karimi et al., 2020
Ethanolic extract of pomegranate	Punicalin, punicalagin, and urolithin A	SARS-CoV-2	Tito et al., 2021; Suručić et al., 2021
Citrus peel extract	Limonene	Influenza A virus H1N1	Fadilah et al., 2022
Citrus peel	Naringin and hesperetin	SARS-CoV-2	Liu et al., 2022; Maulydia et al., 2022
Banana wastes	Alkaloids, flavonoids, tannins, saponins, and glycosides	CHIKV, EV71, and YFV	Panda et al., 2020

Polyphenol extracts from muscadine grape shells, as well as seeds, had a significant bactericidal effect against Gram-positive bacteria like *S. aureus* and *Listeria monocytogenes in vitro*, but only an inhibitory effect against *Salmonella enteritidis* (Gram-negative) (De-Souza et al., 2014; Xu et al., 2016). The variations of antibacterial and bactericidal activities depend on not only the concentration of polyphenolic compounds but also the presence of specific components with different combinations that may result from the synergistic effect those components (Xu et al., 2014). Recent studies reported that hydroethanolic extract of skin/seed or skin-seed mixture has antibacterial activities against both the Gram-positive and harmful bacterial strains such as *E. faecalis, L. monocytogenes, Listeria innocua, E. coli, P. aeruginosa, Klebsiella pneumoniae, Morganella morganii* with MIC ranges from 5 mg/ml to 20+ mg/ml (Peixoto et al., 2018; Gerardi et al., 2021; Gómez-Mejía et al., 2021). Both the seed and pomace extract of grape showed a more potent impact on Gram-positive bacteria (*S. aureus, L. monocytogenes*, and *E. faecalis*) than Gram-negative ones (Table 2; Pfukwa et al., 2019; Gerardi et al., 2021). This is due to the lipopolysaccharide cell wall, which may prevent polyphenols from entering the cell effectively (Gerardi et al., 2021). The inhibitory effect of grape pomace or seed extract substantially correlates with flavonoid, anthocyanin, and proanthocyanidins, potent bioactives of the grape extract (Pfukwa et al., 2019).

Whole grape extracts have been reported to inhibit different enteric viruses and type 1 of HSV (Figure 3; Pascual et al., 2022). Studies reported that grape seed extract had potent antiviral activities against human norovirus surrogates and hepatitis A virus (HAV) (Table 3; Joshi et al., 2015; Oh et al., 2022). Another report suggested that the active compounds from the grape seed extract can block the hepatitis C virus (HCV) replication in HepG2 cells *in vitro* (Pasqua et al., 2016). An *in vitro* study reported that proanthocyanidin, a bioactives from grape seed extract, had anti-viral activity against respiratory syndrome virus and porcine reproductive virus infection in cell line (Zhang et al., 2018). Recently, Chen et al. (2023) demonstrated that grape seed proanthocyanidins extract inhibits dengue virus (DENV) replication by reducing COX-2 expression by modulating NF-κB translocation and ERK/P38 MAPK signaling pathways (Table 3; Chen et al., 2023).

Several bioactive phytochemicals, such flavonoids, phenolics, alkaloids, anthocyanins, and tannins are abundant in pomegranates. It shows a broad spectrum of anti-microbial activity against various pathogenic and multidrug-resistant bacterial strains. Several studies reported that pomegranate peel methanolic extracts had antimicrobial activity against several pathogenic and foodborne bacterial strains like *S. aureus, B. cereus, L. monocytogenes, P. aeruginosa, K. pneumonia, E. coli, Salmonella typhi Bacillus megaterium*, and *B. subtilis* (Table 2; Hasan et al., 2018; Abou El-Nour, 2019; Belgacem et al., 2020; Alsubhi et al., 2022). Whereas, ethyl acetate crude extract exhibited antibacterial activity in plant pathogenic bacteria (*Xanthomonas gardneri, Pectobacterium carotovorum*, and *Ralstonia solanacearum*) (Khaleel et al., 2018).

In the Madin-Darby Canine Kidney (MDCK) cell line-based experiment, pomegranate peels n-butanol and ethyl acetate extract showed an inhibitory action against influenza A virus having IC_50_ value that ranges from 5 to 6 μg/ml (Figure 3; Moradi et al., 2019). According to Houston et al. (2017), punicalagin, a bioactives of pomegranate peel extract, has potent anti-viral activity against HSV-1 (Table 3; Houston et al., 2017). Ethanolic fractions of pomegranate peel containing ellagic acid, punicalagin, and gallic acid were proven effective in anti-adenoviral activity, having IC50 values of 2.16 mg/ml through inhibiting adsorption and post-adsorption phases of viral replication in human epithelial (Hep-2) cell lines (Karimi et al., 2020). Phenolic compounds and tannins from pomegranate peel extract significantly reduce the number of human noroviruses (HuNoV) particles, the causative agent of viral gastroenteritis in different food components (Živković et al., 2021). A recent *in silico* and *in vitro* study reported that ethanolic extract of pomegranate containing punicalin, punicalagin, and urolithin A showed effective inhibition of SARS-CoV-2 through both (Table 3; Tito et al., 2021). The synergistic effects of bioactive substances prevent SARS-CoV-2 spike protein from interacting with human angiotensin-converting (ACE)-2 receptors and limit the viral protease’s activity *in vitro*, ultimately preventing viral multiplication and infection (Figure 3; Suručić et al., 2021).

Disc diffusion test confirmed that the essential oils such as linalool, linalyl acetate, γ-terpinene, narirutin, naringin, limonene, hesperetin-7-o-rutinoside, naringenin, quinic acid, and sakuranetin, identified from *Citrus* spp. showed potent inhibition of *Bacillus* spp., *E. coli*, *E. faecalis, L. monocytogenes*, *B. cereus*, *S. typhimurium*, and *S. aureus*. Limonene is the pre-dominant component contributing to the anti-bacterial potentiality (Table 2; Oikeh et al., 2020; Shehata et al., 2021; Hasan et al., 2022; Cebi and Erarslan, 2023; Meryem et al., 2023; Saleem et al., 2023). Another study reported that in disc diffusion test, the highest inhibitory activity of orange peel oil was demonstrated against Gram-positive *S. aureus*, compared to Gram-negative bacterial strains like *E. coli* (Table 2; Kamel et al., 2022).

Citrus peel extract containing essential oils and limonene (both L and D-limonene) exhibited virucidal activity against influenza A virus H1N1 (Figure 3; Fadilah et al., 2022). Different inflammatory disease conditions, such as SARS-CoV-2 infection was shown to be controlled by the reduction of pro-inflammatory cytokines iNOS, IL-1β, IL-6, and COX-2 expression, with the exposure of citrus peel bioactive compounds (active compound – naringin and hesperetin) both *in vitro* and *in vivo* (Figure 2; Liu et al., 2022). These phytochemicals are the most potent compounds targeting ACE2 receptor for the blockage of SARS-CoV-2, which was further validated through an *in silico* molecular docking approach (Table 3; Maulydia et al., 2022). Moreover, the peels and pulp of *Citrus* contain arbutin, oleanolic acid, malaxinic acid, ursolic acid, chlorogenic acid, epicatechin, and procyanidin B2 that exhibited antibacterial effects (Li et al., 2014; Hussain et al., 2022).

Ethanolic banana peel extract was reported to prevent the growth of *S. typhimurium*, *B. cereus*, *K. pneumoniae, S. aureus*, *L. monocytogenes, Proteus vulgaris, S. pyogenes*, and *E. coli in vitro* (Table 2; Saleem and Saeed, 2020; Hanafy et al., 2021; Maryati et al., 2021; Anandhi and Rajeshkumar, 2023; Singh et al., 2023). β-Sitosterol, malic acid, succinic acid, and palmitic acid from banana peel extract (aqueous/ethyl acetate) showed anti-bacterial activity against food poisoning bacterial strains like *S. aureus, B. subtilis, B. cereus, S. enteritidis*, and *E. coli* (Hikal et al., 2022). Studies also reported that extracts of banana waste products in different solvents, including hexane, acetone, ethanol, and water, showed *in vitro* antiviral activity against the chikungunya virus (CHIKV), enterovirus 71 (EV71), and yellow fever virus (YFV) (Panda et al., 2020).

## Fruit wastes as a source of prebiotic

**FIGURE 4:**
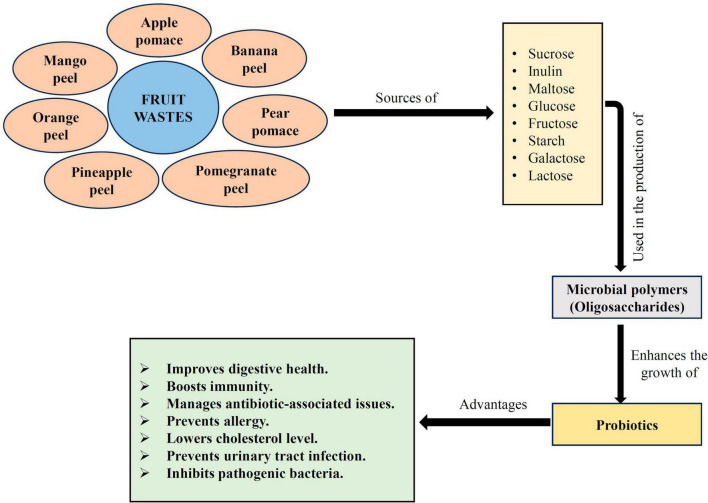
An approach for enhancement of probiotic growth by prebiotic supplementation. Figure generated using Microsoft PowerPoint 2021, 64-bit (Version 2302, Build 16130.20306).

As per FAO/WHO (2002), probiotics are beneficial bacterial strains that improve the host’s health when taken in sufficient amounts. Several studies have been done to increase the growth of probiotics in food and nutritional items by supplementing them with prebiotics, which may be an approach to obtain health advantages. A prebiotic is a selective elements that permits particular changes in the gastrointestinal (GI) tract microflora’s composition and activity, both of which are advantageous to the host’s well-being and health (Gibson et al., 2004). Prebiotic properties are typically present in non-digestible polysaccharides and oligosaccharides, such as galactooligosaccharides, fructooligosaccharides, resistant starch, lactulose, and inulin, that are obtainable from a variety of sources, including fruits and vegetables (Figure 4; Thammarutwasik et al., 2009). A few of them, such as inulin, galactooligosaccharides, and fructooligosaccharides, are major prebiotics marketed for industrial use. A synbiotic product is created when probiotics and prebiotics are combined into a single substrate (Donkor et al., 2007; Sah et al., 2016). Probiotics are consortia of yeasts, molds, or lactic acid bacteria (Sha et al., 2018, 2019). In recent days, industrial microbiologists and food biotechnologists have been very much interested in exploring and introducing new prebiotic compounds with added functional properties, such as fiber-rich fractions from grains, fruits, and vegetables, for a variety of reasons, including industrial significance, and health benefits. Many research works have been conducted on prebiotics to boost the development of probiotic microbes by fortification or enrichment of galactooligosaccharides, fructooligosaccharides, rich herbal fractions (Chowdhury et al., 2008), cereals (Vasiljevic et al., 2007), and passion, banana, apple processing fruit wastes or by-products (Espírito Santo et al., 2012).

## Value addition to fruit wastes

**FIGURE 5:**
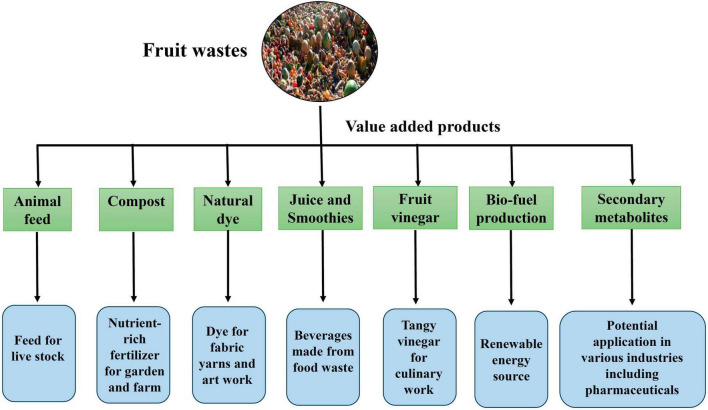
Value addition of various fruit wastes to food, animal feed, energy generation, and medicines production. Figure generated using Microsoft PowerPoint 2021, 64-bit (Version 2302, Build 16130.20306).

Different types of fruit-waste converted into value-added products, including nutritional foods, bioplastics, biosurfactants, bioenergy, biofertilizers, and single-cell proteins, have potential biotechnological significance. A ground-breaking method of decreasing waste and creating new economic opportunities is the value addition of fruit waste through the creation of value-added products based on their bioactive ingredients (Figure 5). These bioactive substances provide numerous health advantages, including polyphenols, vitamins, minerals, and prebiotics and raise the value of the products (Vilas-Boas et al., 2021). It is feasible to produce rich and functional food components, cosmetics, and nutritional supplements using fruit waste’s capacity to extract these substances (Figure 5). This strategy intends to encourage the development of a circular economy while reducing fruit waste and generating new sources of income. Future fruit-waste valorization must consider this waste’s availability throughout time, its techno-economic potential, and the environmental evaluation of benefits and costs based on its life cycle to be both environmentally and economically sustainable (Caldeira et al., 2020). There are various approaches for converting and recycling fruit waste into value-added products for the betterment of human beings (Caldeira et al., 2020).

## Conclusion

The fruit wastes operate as enormous bioactive reservoirs with various health-promoting functions. It has been shown that fruit waste and its bioactives act as potent anti-inflammatory agents and help prevent several cardiovascular disorders by modulating serum cholesterol. Additionally, the secondary byproducts demonstrated their synergistic role in combating hypoglycemic activity and regulate IR. Moreover, this review also supports the anti-viral and anti-bacterial potency of various secondary metabolites derived from fruit waste as well as these fruit waste contains galactooligosaccharides, fructooligosaccharides in rich fractions, regarded as prebiotics that promotes growth of probiotic consortia of human gut and hence, provides various health benefits. Value addition of various fruit wastes into various products like food, animal feed, energy generation, and medicines production can be done for human welfare. Due to the field’s rapid evolution, this review may not fully reflect the most recent developments or newly emerging research on therapeutic applications of fruit waste-derived secondary metabolites in human health. However, this comprehensive review summarizes the current achievements and will enlighten the reuse of various fruit wastes toward sustainable use of these neglected bioactive chemicals and by-products in diverse biological and pharmacological applications that might help the world attain its “zero waste” objective and human welfare.

## Author contributions

SSh: Conceptualization, Writing – review and editing, Supervision. SB: Conceptualization, Writing – review and editing. KG: Conceptualization, Writing – review and editing. DM: Validation, Writing – review and editing. SS: Writing – review and editing. SSa: Validation, Writing – review and editing. SR: Formal analysis, Writing – review and editing.
